# Targeted sequencing of 351 candidate genes for epileptic encephalopathy in a large cohort of patients

**DOI:** 10.1002/mgg3.235

**Published:** 2016-07-30

**Authors:** Carolien G.F. de Kovel, Eva H. Brilstra, Marjan J.A. van Kempen, Ruben van‘t Slot, Isaac J. Nijman, Zaid Afawi, Peter De Jonghe, Tania Djémié, Renzo Guerrini, Katia Hardies, Ingo Helbig, Rik Hendrickx, Moine Kanaan, Uri Kramer, Anna‐Elina E. Lehesjoki, Johannes R. Lemke, Carla Marini, Davide Mei, Rikke S. Møller, Manuela Pendziwiat, Hannah Stamberger, Arvid Suls, Sarah Weckhuysen, EuroEPINOMICS RES Consortium, Bobby P.C. Koeleman, Balling R, Barisic N, Baulac S, Caglayan HS, Craiu DC, Depienne C, Gormley P, Hjalgrim H, Hoffman‐Zacharska D, Jähn J, Klein KM, Komarek V, LeGuern E, Lerche H, May P, Muhle H, Pal D, Palotie A, Rosenow F, Selmer K, Serratosa JM, Sisodiya SM, Stephani U, Sterbova K, Striano P, Talvik T, van Haelst M, Verbeek N, von Spiczak S, Weber YG

**Affiliations:** ^1^ Department of GeneticsUMC UtrechtUtrechtThe Netherlands; ^2^ Tel Aviv Sourasky Medical Center6 Weizmann St.Tel AvivIsrael; ^3^ Genetics of Epilepsy Research in Israel Tel‐Aviv University Medical SchoolTel‐AvivIsrael; ^4^ Neurogenetics GroupDepartment of Molecular GeneticsVIBAntwerpBelgium; ^5^ Laboratory of NeurogeneticsInstitute Born‐BungeUniversity of AntwerpAntwerpBelgium; ^6^ Division of NeurologyAntwerp University HospitalAntwerpBelgium; ^7^ Neuroscience DepartmentChildren's Hospital Anna MeyerUniversity of FlorenceFlorenceItaly; ^8^ Division of NeurologyThe Children's Hospital of PhiladelphiaPhiladephiaPennsylvania; ^9^ Department of NeuropediatricsUniversity Medical Center Schleswig‐HolsteinChristian Albrechts UniversityKielGermany; ^10^ Pediatric Epilepsy UnitTel Aviv Sourasky Medical CenterTel Aviv UniversityTel AvivIsrael; ^11^ Department of Life SciencesBethlehem UniversityBethlehemPalestine; ^12^ Folkhälsan Institute of GeneticsNeuroscience Center and Research Programs UnitMolecular NeurologyUniversity of HelsinkiHelsinkiFinland; ^13^ Institute of Human GeneticsUniversity of Leipzig Hospitals and ClinicsLeipzigGermany; ^14^ Danish Epilepsy Centre – FiladelfiaDianalundDenmark; ^15^ Institute for Regional Health ServicesUniversity of Southern DenmarkDK‐5230OdenseDenmark

**Keywords:** *De novo*, epileptic encephalopathy, *HNRNPU*, loss‐of‐function, prioritization, recessive, targeted panel sequencing, X‐linked

## Abstract

**Background:**

Many genes are candidates for involvement in epileptic encephalopathy (EE) because one or a few possibly pathogenic variants have been found in patients, but insufficient genetic or functional evidence exists for a definite annotation.

**Methods:**

To increase the number of validated EE genes, we sequenced 26 known and 351 candidate genes for EE in 360 patients. Variants in 25 genes known to be involved in EE or related phenotypes were followed up in 41 patients. We prioritized the candidate genes, and followed up 31 variants in this prioritized subset of candidate genes.

**Results:**

Twenty‐nine genotypes in known genes for EE (19) or related diseases (10), dominant as well as recessive or X‐linked, were classified as likely pathogenic variants. Among those, likely pathogenic *de novo* variants were found in EE genes that act dominantly, including the recently identified genes *EEF1A2, KCNB1* and the X‐linked gene *IQSEC2*. A *de novo* frameshift variant in candidate gene *HNRNPU* was the only *de novo* variant found among the followed‐up candidate genes, and the patient's phenotype was similar to a few recent publications.

**Conclusion:**

Mutations in genes described in OMIM as, for example, intellectual disability gene can lead to phenotypes that get classified as EE in the clinic. We confirmed existing literature reports that *de novo* loss‐of‐function *HNRNPU*mutations lead to severe developmental delay and febrile seizures in the first year of life.

## Introduction

According to the ILAE definition epileptic encephalopathy (EE) is a condition in which “the epileptiform electroencephalographic (EEG) abnormalities themselves are believed to contribute to a progressive disturbance in cerebral function” (Engel and International League Against 2001). It is a highly heterogeneous disorder, with variation in the age at onset, the type and distribution of seizures, developmental outcome,EEG patterns, the response to medication, and a wide range of comorbidities. Genetically, it is just as heterogeneous, and OMIM currently lists 32 genes recognized for early infantile EE (EIEE), with different inheritance modes (data accessed August 2015). Apart from this list, patients who are diagnosed with EE regularly turn out to have a pathogenic variant in a gene that is annotated for intellectual disability with seizures (Hirose and Mitsudome 2003). With the current list of genes associated with EE, a genetic diagnosis in the clinic can be made in ~10‐15% of referred patients (pers. comm. Marjan van Kempen). Since in the majority of these patients a variant of large effect is expected, this means many genes that may cause EE when mutated probably remain to be discovered.

Several recent efforts have been undertaken to identify additional EE genes. For example, the Epi4K effort sequenced 264 parent‐offspring trios of patients with infantile spasms or Lennox–Gastaut syndrome using whole exome sequencing (WES) (Epi4K‐Consortium et al. 2013; Consortium et al. 2014). They identified on average 1.25 *de novo* variants per person in those patients. Yet, only nine novel genes showed a significant excess of *de novo* variants and could be classified as EE genes, explaining the condition in 29 patients. For the genes with one or two *de novo* hits in the whole cohort, the status remained unclear. Other efforts using whole exome sequencing or targeted sequencing (Carvill et al. 2013; Kodera et al. 2013; Ohba et al. 2013; Veeramah et al. 2013; Consortium et al. 2014) have identified a number of genes enriched for variants in patients with EE. Yet, also in these efforts, *de novo* variants in many candidate genes were not observed to be significantly overrepresented in patients, possibly due to small numbers. The number of *de novo* hits required to confidently link a gene to a specific disease depends on the number of patients involved in studies and gene size, as well as on supportive evidence from clinical (similarity among patients with variants in the same gene) and/or functional studies. Recessive diseases are often, though not always, caused by inherited variants, and even more stringent criteria for pathogenicity should be applied. In many recent publications homozygous or compound heterozygous hits have been found in two or three sibs only (Simpson et al. 2004; Banne et al. 2013; Basel‐Vanagaite et al. 2013; Paciorkowski et al. 2014), which is not statistically convincing. Here too, additional support is required.

In our current study, we aimed to increase the number of known genes for EE by confirming the involvement of candidate genes from previous studies using targeted resequencing in 359 unrelated patients. As the majority of previous studies focused on genes that cause EE in a dominant fashion, we included genes that influence EE in an X‐linked or recessive fashion with priority. We also preferentially included probands from consanguineous and multiplex families. We included known EE genes in the design because patients were not previously tested exhaustively for all known genes. Thus, we could sort results into results for patients with known causes and those for whom a novel cause would be more likely. We present our results for the cohort of 359 independent patients.

## Methods

### Samples

Patients included in this study had an onset of seizures and concomitant intellectual disability (ID) before the age of five years, and no pathogenic variants had been identified in previous diagnostic testing. Samples were collected by multiple centers, and previous genetic testing had been carried out following local guidelines. The majority of patients were European, but at least 68 patients were Arab, Turkish, or North‐African, according to their parents' specification. The parents of 209 patients collected in the Netherlands, Belgium, France, Germany, Denmark, and Bulgaria were not specifically asked about their ethnicity. Of these, at least 17 were likely non‐European based on family name; the others were likely in majority of European descent.

In total, 359 samples were included including two sibs. Sixty‐five probands had affected family members, mainly sibs, who were not included in the study. In most of these patients, there was no known relationship between the parents. Forty‐one patients were sporadic cases, but with related parents. The patients with affected sibs would likely have a recessive inheritance, since parents were not suffering from EE. However, the possibility of parental mosaicism cannot be neglected. The sporadic patients with related parents are likely enriched for recessive inheritance as well. We had 155 females and 204 males. In this article, we will refer to our dataset as “TEGA” (Targeted Epilepsy Genes Array).

### Ethical compliance

Ethical approval and consent were obtained at the local institutions. Collection of patients was reviewed and approved by local ethics committees.

### Sequencing and mapping

Genes were collected up to a footprint of ~ 1.2 Mb. We collected genes from the following studies: (Simpson et al. 2004; Striano et al. 2007; Backx et al. 2009; Janer et al. 2012; Banne et al. 2013; Basel‐Vanagaite et al. 2013; Carvill et al. 2013; Frühmesser et al. 2013; Kodera et al. 2013; Ohba et al. 2013; Veeramah et al. 2013; Consortium et al. 2014; Paciorkowski et al. 2014),and from our own earlier experiments. More information on many of the genes may also be found on: http://122.228.158.106/EpilepsyGene/index.php.We included known genes for EIEE from OMIM as well. This resulted in 273 genes that are described as influencing EE phenotypes in an autosomal dominant fashion, 68 that act as autosomal recessive, 2 not clear, 26 X‐linked, and 1 mitochondrial genes. A list of genes and some characteristics, for example, expected mode of inheritance and reason for inclusion can be found in Table S1. In August 2015, twenty‐seven genes in our design were listed in OMIM as genes causing EE or very similar phenotypes (128,435 bp); 25 of our genes were annotated with phenotypes that included mental retardation or metabolic disorders, but had been found mutated in EE patients before (143,815 bp). The remaining 290 genes were not annotated for a suspect phenotype (889,391 bp), though a few are associated with GEFS+ or other milder epilepsy phenotypes (Table S1).

Probes for enrichment were designed and ordered from Agilent Technologies (Santa Clara, CA, USA) as Sure Select enrichment kit. Experimental workflow followed the standard protocols. Sequencing was done on a SOLiD5500XL. Alignment, variant calling, and annotation were done using an in‐house pipeline, which used a BWA‐aligner and a GATK‐based variant caller versus Human reference GhCr37/hg19 (McKenna et al. 2010). Aligned data were processed following the Genome Analysis Toolkit (GATK) best practices v2. Reads were realigned around indels, using GATK IndelRealigner, and base quality scores were recalibrated, using GATK BaseRecalibrator. Annotation was based on Ensembl GRCh37.p13 and dbSNP 144.

### Quality control and filtering

Variants were included if they had at least 10x coverage, an alternative variant call of at least 20%, and were annotated as affecting the protein. Variants that were seen in more than eight additional samples per plate at low percentages of variant alleles (< threshold of 20%) were excluded, since these probably constitute noise. Positions that showed a third allele at >4% of reads averaged over all samples per plate were also excluded, as probable artifacts. Positions that had failed in more than 10% of the samples were only included after visual inspection.

For dominant genes, we included only variants not listed in dbSNP v144, on the ESP server (http://evs.gs.washington.edu/EVS/ May 2015) or the ExAC database (http://exac.broadinstitute.org, June 2015). These variants we annotated as “novel.” We added variants that were annotated as “of clinical significance” in dbSNP, if this annotation referred to epilepsy or epilepsy‐related disorders. For autosomal and X‐linked recessive genes as well as for the female‐specific X‐lined gene *PCHD19* (MIM 300460), we added variants that were annotated as having a population frequency of at most 0.001. We checked that no homozygotes or X‐linked hemizygotes of this variant were observed in the databases ESPor ExAC (except for *PCDH19*). Variants that were seen in more than two patients in our panel of 359 independent samples were excluded.

### Follow‐up

Variants were annotated with their CADD‐score (Kircher et al. 2014), SIFT (Kumar et al. 2009; Hu and Ng 2012), Polyphen‐2 (Adzhubei et al. 2013), Grantham (Grantham 1974) and Gerp‐score (Cooper et al. 2005) (when available), and the T/D annotation of MetaLR (Dong et al. 2015). Variants in known genes for EE were Sanger‐sequenced in the patient as well as in the parents if DNA was available (*N* = 41). Clinical experts assessed whether causality was likely based on previously published phenotypes for these genes, and the clinician's experience. Patients with a pathogenic variant in a known EE gene were flagged. Variants in a subset of candidate genes were followed‐up (*N* = 31), as explained below.

### Prioritization

To prioritize genes for follow‐up, we wanted to identify genes with an excess of novel variants in our patients. The frequency of unique variants in our patients was compared to the frequency in the ExAC database as follows.

For all genes on our design, the coding variants were downloaded from the ExAC‐database, along with the number of alleles that were seen in the database at that position. We combined these data with our results and selected those variants that were seen only once in this combined dataset. The frequency of unique variants was compared between the datasets with a Fisher exact test. We ranked the genes with excess variation in our dataset according to the *P*‐value.

We selected a subset of candidate genes for follow‐up based on ranking in our excess‐list and preliminary data of parallel studies of consortium members. For this subset of variants, we used Sanger sequencing in DNA samples from the proband and the parents – if available – to assess whether the inheritance pattern confirmed our expectation, i.e. *de novo* for heterozygous variants in dominant‐acting genes; homozygous, compound heterozygous, or *de novo* for double hits in autosomal recessive‐acting genes; *de novo* or inheritance from mother in X‐linked recessive‐acting genes; *de novo* or inheritance from healthy father or affected mother for *PCDH19*.

To get more insight, we compared the unique variants listed in ExAC with the unique variants found in our patients on the CADD score for deleteriousness. The CADD‐score incorporates a large number of other scores, and was designed to identify deleterious variants rather than, for example, variants with any effect on the protein functioning. We also compared CADD‐scores of *de novo* variants in dominant‐acting EE‐genes with CADD‐scores of inherited variants.

### Statistics

To test whether known EE genes were overrepresented among the genes with excess novel variants we used a chi‐square test for independence. This test was also used to test whether recessive disorders were overrepresented among children from related parents, and to test whether LoF variants were overrepresented in genes that were classified as intolerant to LoF.

To compare CADD‐scores between groups of genes or groups of variants, we performed ANOVA tests on the raw CADD‐scores, rather than the phred‐scores, as their distribution was closer to normal.

To compare the number of unique variants per gene in our patients versus the ExAC database, we used Fisher exact tests, since cell counts were often small. Because of differences in data collection, a good threshold for significance is hard to set. A conservative approach would be alpha = (0.05/361) = 1.4e‐4. In our analysis, *P*‐values were simply used to rank the genes.

As a threshold for significance for the other tests, we used alpha = 0.01.

## Results

One sample failed completely (median coverage < 1x). Fourteen other samples had less than 70% of the target covered at 20x, but they were still included in the analysis. Median coverage of the included samples was 114x (SD 57), complexity 45 (SD 14). Genes in our design were on average covered at > 10x for 94.5% (SD 8) of their length. *MROH8* was covered for 35% and *BHLHE22* not at all.

In the remaining cohort of 358 independent samples, we identified in total 388 novel variants, i.e. not in dbSNP 144, ESP server or ExAC database, varying from 0–8 per sample, single heterozygous variants in genes with a presumed recessive effect on EE excluded. Of those variants 18 were frameshift indels, 5 were in‐frame indels, and the remainder were substitutions, which included 13 nonsense, one splice donor site and three splice acceptor sites. Five variants occurred in two independent samples each. Eighty‐eight variants were found in genes annotated as EE (55) or with related OMIM‐annotations (33). An overview can be found in supp. Table S1 and full list in Table S2.

Patients of North‐African or Middle Eastern descent carried on average 1.6x as many novel variants per individual as patients from European descent (Poisson, *P *=* *0.0002). A likely explanation is that the public databases contain an excess of European samples, so polymorphisms specific to other geographical regions are underrepresented.

### Known EE genes and genes for related phenotypes

Forty‐one variants in 25 genes with an OMIM annotation for EE, ID or related phenotype were followed up. By investigating the segregation pattern and investigating the phenotype, we found presumably pathogenic hits in known genes in 29 patients: 20 in EE genes, and nine in genes for related phenotypes (Table I in Table S1). Heterozygous variants with a presumed dominant effect were either novel or only seen in other patients, and they were *de novo*. For the presumed recessive variants, they were either earlier reported as pathogenic and very rare (<1:10,000 in ExAC), or novel, and the treating clinician reported that the patient's symptoms fit with gene, from literature reports and their own experience. Exception is presumed recessive rs376712059 in *TBC1D24*, which is very rare and reported in ClinVar as “With Uncertain significance allele”; the other variant in this gene is this patient was reported as pathogenic before (Campeau et al. 2014). The clinician was convinced that this was a very probable causal gene for this patient. In *PNKP* homozygous LoF variants have been reported as pathogenic before (Poulton et al. 2013), which is why we considered this homozygous frame shift mutation as pathogenic. In all patients listed in Table 1, the clinicians reported that the gene was a likely fit for the patients' symptoms. Though prediction scores were not used to assess pathogenicity, all variants in this Table have CADD phred‐ scores >23.0.

**Table 1:** Probably pathogenic variants confirmed in follow‐up

ID	Sex	Genotype	Gene	Variant (aa)^a^	Expected gene inheritance	Family tested	OMIM morbid description^b^
2012D09029	M	Hemi	*CASK*	p.R584X	X‐linked	De novo	Mental retardation and microcephaly with pontine and cerebellar hypoplasia
2008D06721	F	Hetero	*EEF1A2*	p.G70S	Dominant	De novo	Epileptic encephalopathy, early infantile, 33
EG1761	F	Hetero	*FARS2*	p.T156M	Recessive	Compound het	Combined oxidative phosphorylation deficiency 14
EG1761	F	Hetero	*FARS2*	c.905‐1G>A (splice‐acceptor)	Recessive	Compound het	Combined oxidative phosphorylation deficiency 14
EP2201	F	Hetero	*GNAO1*	p.G40R	Dominant	De novo	Epileptic encephalopathy, early infantile, 17
EP2822	M	Hetero	*GPHN*	p.D422N	Recessive	Compound het	Molybdenum cofactor deficiency, complementation group c
EP2822	M	Hetero	*GPHN*	c.1315‐2A>G (splice‐acceptor)	Recessive	Compound het	Molybdenum cofactor deficiency, complementation group c
EP1718	M	Homo	*GRIN1*	p.Q556X	Dominant	Inherited from het parents	Mental retardation, autosomal dominant 8
EP2797	M	Hetero	*GRIN1*	p.G827R	Dominant	De novo	Mental retardation, autosomal dominant 8
2012D06376		Hetero	*HNRNPU*	pV604 fs	Dominant	De novo	*Candidate*
2010D12136	M	Hemi	*IQSEC2*	p.Y1129X	X‐linked	De novo	Mental retardation, x‐linked 1
EP1961	F	Hetero	*IQSEC2*	p.G771D	X‐linked	De novo	Mental retardation, x‐linked 1
2010D05815	F	Hetero	*KCNB1*	p.F416L	Dominant	De novo	Epileptic encephalopathy, early infantile, 26
KIEL20	M	Hetero	*KCNB1*	p.R312H	Dominant	De novo	Epileptic encephalopathy, early infantile, 26
2012D20026	M	Hetero	*KCNQ2*	p.Y363H	Dominant	De novo	Epileptic encephalopathy, early infantile, 7
KIEL42	F	Hetero	*KCNQ2*	p.R532W	Dominant	De novo	Epileptic encephalopathy, early infantile, 7
2009D12616	F	Hetero	*KCNT1*	p.R429C	Dominant	Not tested	Epileptic encephalopathy, early infantile, 14
EP2788	F	Hetero	*KCNT1*	p.R429H	Dominant	De novo	Epileptic encephalopathy, early infantile, 14
EP95	M	Hetero	*KCNT1*	p.R429C	Dominant	De novo	Epileptic encephalopathy, early infantile, 14
1011L	F	Homo	*PNKP*	p.A420 fs	Recessive	Inherited from het parents	Epileptic encephalopathy, early infantile, 10
395M	F	Homo	*POLG*	p.R1096C	Recessive	Inherited from het parents	Leigh syndrome
EP1781	F	Hetero	*SCN1A*	p.C968G	Dominant	De novo	Epileptic encephalopathy, early infantile, 6
EUR577	F	Hetero	*SCN1A*	p.I1347T	Dominant	De novo	Epileptic encephalopathy, early infantile, 6
KIEL38	M	Hetero	*SCN1A*	p.D702 fs	Dominant	De novo	Epileptic encephalopathy, early infantile, 6
EP1789	F	Hetero	*SCN2A*	p.L1665F	Dominant	De novo	Epileptic encephalopathy, early infantile, 11
EP2104	M	Hetero	*SCN2A*	p.Q1811E	Dominant	De novo	Epileptic encephalopathy, early infantile, 11
2010D14438	F	Hetero	*SLC13A5*	p.S427L	Recessive	Compound het	Epileptic encephalopathy, early infantile, 25
2010D14438	F	Hetero	*SLC13A5*	p.G219R	Recessive	Compound het	Epileptic encephalopathy, early infantile, 25
EP2821	F	Homo	*SLC25A22*	p.Q117R	Recessive	Inherited from het parents	Epileptic encephalopathy, early infantile, 3
EP2806	F	Hetero	*SPTAN1*	p.R2037W	Dominant	De novo	Epileptic encephalopathy, early infantile, 5
EP2514	M	Hetero	*STXBP1*	p.P480L	Dominant	De novo	Epileptic encephalopathy, early infantile, 4
2013D03222	M	Double het	*TBC1D24*	p.E153K	Recessive	Testing	Epileptic encephalopathy, early infantile, 16
2013D03222	M	Double het	*TBC1D24*	p.H336 fs	Recessive	Testing	Epileptic encephalopathy, early infantile, 16
2008D07479	F	Hetero	*WDR45*	p.E155X	X‐linked dominant	De novo	Neurodegeneration with brain iron accumulation 5; SENDA

Compound heterozygotes are shaded in gray.

a Complete notation with accession numbers can be found in the supplementary table.

b Morbid description in OMIM. Only the most relevant phenotype is listed here.

In sporadic patients with presumably unrelated parents, our follow‐up experiments detected fifteen *de novo* variants in autosomal dominant‐acting genes; four *de novo* variants in X‐linked genes, and three compound heterozygous variant combinations in recessive‐acting genes. One of those compound heterozygous combinations was also detected in an affected sib. In patients with related parents, we detected three homozygous variants in recessive genes, one homozygous variant in presumed dominant gene *GRIN1* (MIM 138249) and one compound heterozygous variant combination in a recessive gene. Though patients with related parents are presumed to be predisposed for recessive‐acting variants, other inheritance mechanism cannot be excluded. One patient with related parents had a *de novo* variant. Heterozygous loss of function variants in X‐linked gene *WDR45* have been reported as enriched in girls with EE‐like phenotypes (Saitsu et al. 2013). We found an interesting in‐frame indel in *WDR45* (MIM 300526) hemizygous in a boy, though its significance remains to be investigated. A complete list of all novel variants and possibly pathogenic variants in recessive genes can be found in Table S2.

We like to highlight a few interesting genes: We found four patients with novel missense variants in *SCN1A* (MIM182389) (Claes et al. 2001), and one with a frameshift variant. Of those, three were *de novo*, while two were inherited, so probably not pathogenic. In addition, one patient had a highly conserved splice region variant, which had been seen in a Dravet patient before as a *de novo* variant (Harkin et al. 2007).

Of special interest were the X‐linked and recessive genes. We found a *de novo* heterozygous nonsense mutation in X‐linked gene *WDR45* (Haack et al. 2012; Saitsu et al. 2013; Ohba et al. 2014; Ozawa et al. 2014) in a girl, but also, as mentioned above, an in‐frame indel in a boy. This gene is described as influencing EE‐phenotypes in an X‐linked dominant fashion. The inheritance in the boy will be investigated, but we currently consider it as a variant of unknown significance.(Haack et al. 2012). In *IQSEC2* (MIM 300522) (Shoubridge et al. 2010; Epi4K‐Consortium et al. 2013; Gandomi et al. 2014; Tran Mau‐Them et al. 2014), we found a hemizygous *de novo* variant in a boy, and a heterozygous *de novo* variant in a girl (Morleo et al. 2008; Olson et al. 2015). Though *IQSEC2*is described in OMIM as acting in a dominant fashion, we considered both variants as pathogenic (Kalscheuer et al. 2015).

### Prioritizing and variant characteristics

We hypothesized that ranking the frequency of unique hits per gene relative to the ExAC database was the most relevant variable for prioritizing candidate genes, despite some caveats (see Discussion). None of the genes showed significant excess in EE cases (Fisher exact, Bonferroni correction). Among the genes with excess novel variants (OR > 1.0) were relatively more known EE and epilepsy‐related genes than among genes with fewer novel variants (*P *<* *0.0001; see Table S3), and in the top ten, six genes were EE or epilepsy‐related. We selected a shortlist of candidate genes for follow‐up. Part of those genes were selected because they were top‐ranking in the list with excess unique variants, while a few others were added because of preliminary data of independent studies of some of the investigators. Our follow‐up list can be found in Table S1.

**Figure 1:**
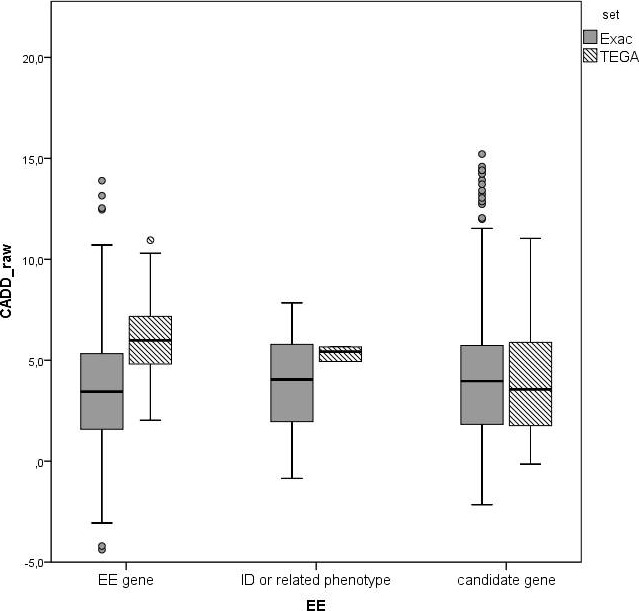
Raw CADD‐scores for novel unique variants in our patient cohort versus unique variants in the ExAC database in three gene categories. (In all figures: note that most indels get no CADD‐scores).

We divided the genes with excess novel variants (OR > 1) in (1) known EE genes, (2) genes in OMIM with intellectual disability or with an epilepsy‐related phenotype, (3) candidate genes without known relatedness to epilepsy. For these three sets, we compared CADD‐scores for unique variants in our patients with CADD‐scores of unique variants in the ExAC database. We found an interaction effect (*P *=* *0.002), where in the EE genes, the CADD‐scores in our patients were higher than in the ExAC database, while this difference was not visible in the candidate genes (Fig. 1).

**Figure 2:**
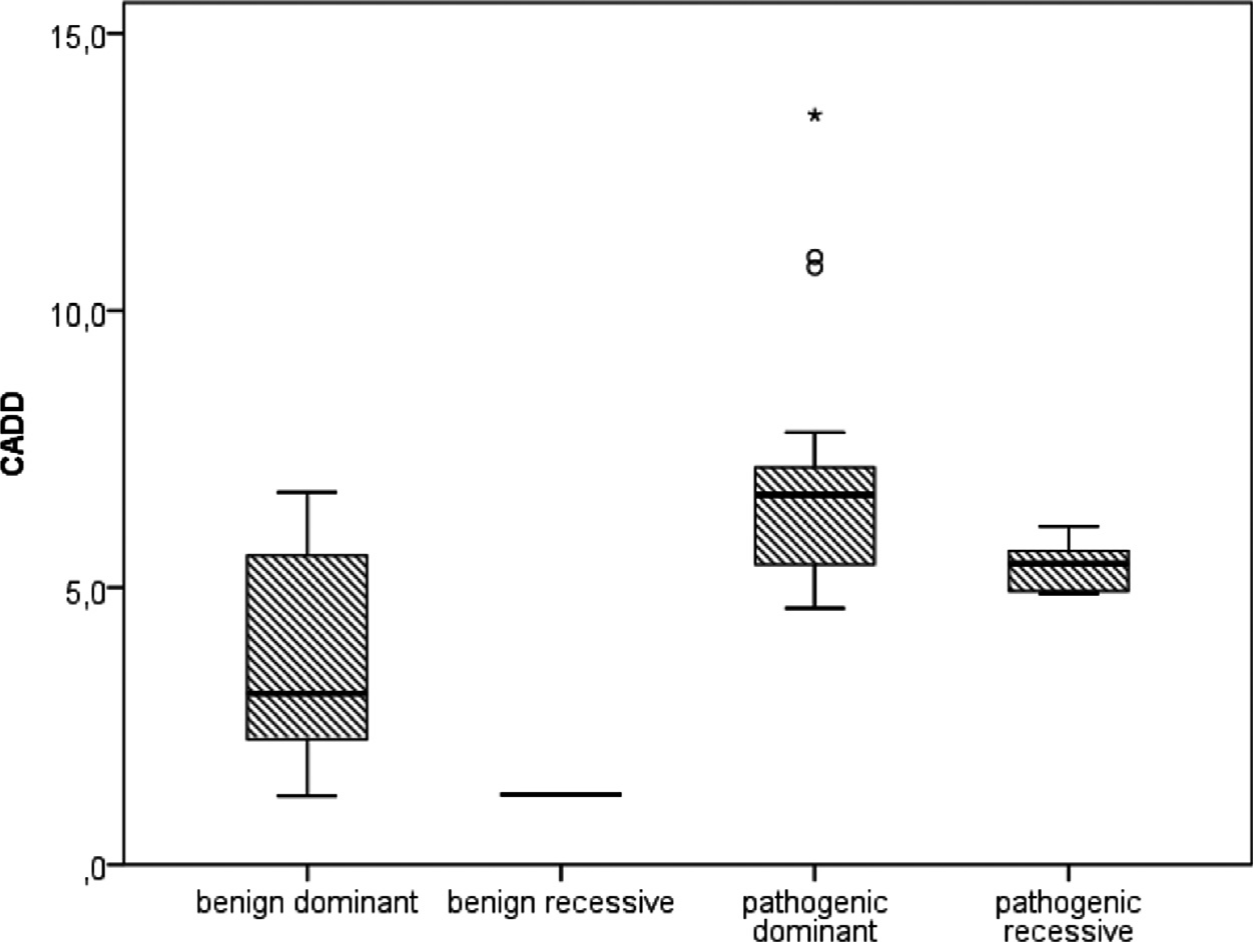
Raw CADD‐scores for novel variants in our patient cohort in known genes for EE or related phenotypes. After checking the variants in relatives, and considering the patient's phenotype, variants were classified into probably benign and probably pathogenic. Recessive‐ and dominant‐acting genes are shown separately.

In the known EE genes, we compared CADD‐scores for variants that were eventually considered pathogenic, based on inheritance and phenotype, with variants that were considered benign, such as inherited variants in dominant genes. The presumed pathogenic variants, both in dominant and recessive‐acting genes had on average higher CADD‐scores than the presumed benign variants (*P *=* *5.7 10^−6^, Fig 2), supporting their involvement in disease.

**Figure 3:**
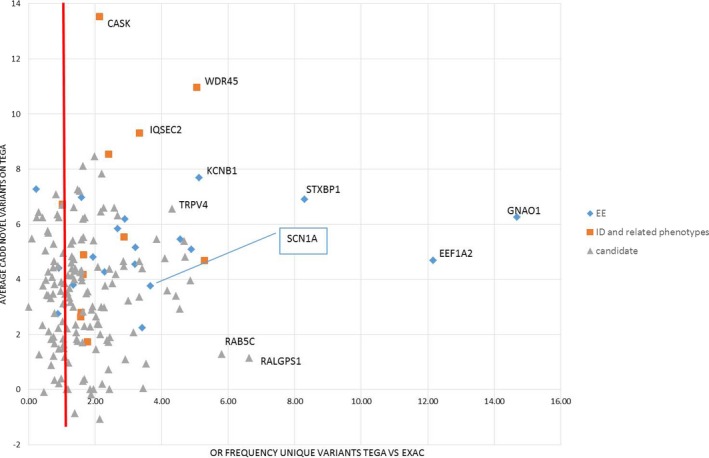
Per gene: Excess frequency of novel variants in TEGA versus ExAC database (*X*‐axis) versus average CADD‐scores for novel variants in TEGA samples (*Y*‐axis). Genes are classified into known EE genes, genes for intellectual disability, or phenotypes that may present with seizures, and candidate genes.

Because both excess unique variants and high CADD‐scores seem to be correlated with gene pathogenicity, we plotted both, to identify promising genes (Fig. 3).

In addition to excess and CADD‐scores, we looked at loss‐of‐function (LoF) variants. In our dataset, we found 34 possibly suspect LoF variants, of which 31 novel and three rare in a recessive gene (Table 2). Eighteen of those were in genes that are intolerant to loss of function variants according to Samocha et al.'s pLi‐score (pLi> 0.95) (Samocha et al. 2014). LoF variants in our dataset occurred more often in intolerant genes than expected by chance (*P *<* *10^−5^). When restricted to the candidate genes only, the excess was less pronounced, but still suggestively significant (one‐sided *P *=* *0.025). Most of those genes, though, also had some LoF‐variants in the ExAC‐database (Table 2), and careful inspection of the position and consequence of all LoF variants in the gene is needed to assess the possible impact of the variants found in our experiment. Clearly not all of those variants should be considered the main cause for the patient's symptoms.

**Table 2:** Novel or very rare loss of function variants

ID	Variant (hg19)	Genotype	Gene	Variant (aa)^a^^,^ b ^,^ c	Expected gene inheritance	Gene pLI^d^ (Samocha)	No. LoF per gene in ExAC
EP949	chr12:123433309‐T/‐	Hetero	*ABCB9*	p.N305 fs*35	Dominant	0.00	7
KIEL99	chr17:48736728‐C/T	hetero	*ABCC3*	p.R269X	Dominant	0.00	30
EUR578	chr19:13563750‐GGAAGGC/‐	Hetero	*CACNA1A*	p.A158 fs*6	Dominant	**1.00**	4
2012D09029	chrX:41414858‐G/A	Hemi	*CASK*	p.R584X	X‐linked	**1.00**	1
2007D04829	chr15:93527629‐TCAT/‐	Hetero	*CHD2*	p.I1046 fs*8	Dominant	**1.00**	5
2010D08930	chr15:93563370‐C/T	Hetero	*CHD2*	p.R1679X	Dominant	**1.00**	5
2008D06063	chr22:38694137‐G/‐	Hetero	*CSNK1E*	p.257 fs (minor transcripts)	Dominant	**0.97**	1
2012D20026	chrX:96603116‐G/C	Hemi	*DIAPH2*	c.2847‐1G>C (minortranscripts, missense in other rs775057363)	X‐linked	**1.00**	3
EUR585	chr3:132235289‐TT/‐	Hetero	*DNAJC13*	p.L1837 fs*48	Dominant	**1.00**	8
EG1761	chr6:5545412‐G/A	Hetero	*FARS2*	c.905‐1G>A (splice‐acceptor)	Recessive	0.00	10
D03/4526	chr11:134175014‐A/‐	Hetero	*GLB1L3*	p.A294 fs*2 (minor transcripts)	Dominant	0.00	22
D04/1316	chr11:134183917‐GA/‐	Hetero	*GLB1L3*	p.E555 fs*50	Dominant	0.00	22
2006D07509	chr16:56226254‐T/G	Hetero	*GNAO1*	p.L36X	Dominant	**0.98**	0
EP2822	chr14:67578576‐A/G	Hetero	*GPHN*	c.1315‐2A>G (splice‐acceptor)	Recessive	**1.00**	1
KIEL38	chr19:6731282‐G/T	Hetero	*GPR108*	p.Y454X	Dominant	0.00	16
EP1718	chr9:140056657‐C/T	Homo	*GRIN1*	p.Q556X	Dominant	**0.97**	4
2012D06376	chr1:245019802–/A	Hetero	*HNRNPU*	pV604 fs *24	Dominant	**1.00**	1
2010D12136	chrX:53265568‐G/T	Hemi	*IQSEC2*	p.Y1129X	X‐linked	**0.98**	1
EP1852	chr20:47990498‐G/T	Hetero	*KCNB1*	p.Y533X	Dominant	**0.98**	1
2012D18530	chr15:52664419‐T/A	Hetero	*MYO5A*	p.K907X	Dominant	**0.99**	16
EUR574	chr2:206617582‐G/T	Hetero	*NRP2*	p.G643X	Dominant	0.00	11
D04/2814	chr5:140603538‐G/‐	Hetero	*PCDHB14*	p.M154 fs*42	Dominant	0.00	12
2012D20026	chr3:126723726‐A/G	Hetero	*PLXNA1*	c.1620‐2A>G (splice‐acceptor)	Dominant	**1.00**	5
1011L	chr19:50365068–/CGACC	Homo	*PNKP*	p.A420 fs*49 (rs768847609)	Recessive	0.00	13
KIEL92	chr17:40278712‐C/T	Hetero	*RAB5C*	p.W130X (minor transcripts)	Dominant	0.83	1
KIEL38	chr2:166898868‐AAAGT/‐	Hetero	*SCN1A*	p.D702 fs*25	Dominant	**1.00**	2
2010D14485	chr20:1293995–/C	Hetero	*SDCBP2*	p.124‐125‐fs*33	Dominant	0.41	2
2006D07509	chr17:80218938–/A	Hetero	*SLC16A3*	p.296 fs (minor transcript)	Dominant	0.33	2
KIEL92	chr5:168123348‐G/‐	Hetero	*SLIT3*	p.T1017 fs*24	Dominant	**0.99**	11
EP2103	chr4:99064223‐G/A	Hetero	*STPG2*	p.Q27X	Dominant	0.00	18
2013D03222	chr16:2548263‐T/‐	Hetero	*TBC1D24*	p.H336 fs*11	Recessive	0.00	10
D02/2287	chr6:30123503‐C/T	Hetero	*TRIM10*	c.928 + 1G>A (splice‐donor)	Dominant	0.00	10
EP2805	chr4:39257574‐T/G	Hetero	*WDR19*	p.Y1036X	Dominant	0.00	20
2008D07479	chrX:48933578‐C/A	Hetero	*WDR45*	p.E155X	X‐linked dominant	**0.97**	0

Bold values indicate significant intolerance score.

a Frameshift consequences calculated with SIFT (Hu and Ng 2012). Nonsense‐mediated decay predicted for all frameshifts except chr19:50365068–/CGACC, chr11:134175014‐A/‐ and chr22:38694137‐G/‐.

b Some variants occur only in less well supported transcripts (“minor transcripts”).

c Full description of variants including accession number in Tables S1–S3.

d Loss‐of‐function intolerance score according to Samocha et al. (2014). Score ranges 0–1, with high scores meaning less tolerant.

### Follow‐up of variants in candidate genes

We followed up 31 patients in the prioritized candidate genes. In the recessive candidate genes, double heterozygote or homozygote genotypes were rare (Table S1). All variants in candidate genes turned out to be inherited, except one: in the gene *HNRNPU* (MIM 602869) we identified a *de novo* mutation (Table I in Table S2). Like other recently published pathogenic variants in *HNRNPU* (Need et al. 2012; Carvill et al. 2013; Epi4K‐Consortium et al. 2013; Hamdan et al. 2014), (https://decipher.sanger.ac.uk/ddd#research-variants/snvs, http://www.ncbi.nlm.nih.gov/clinvar/?term=hnrnpu[gene]), this variant causes termination of the transcript. Nonsense mediated decay is a possible consequence. The gene is located within the region 1q43q44 where a recurrent microdeletion is found, which has been detected in patients with intellectual disability, microcephaly, craniofacial anomalies, seizures, limb anomalies, and corpus callosum abnormalities (Caliebe et al. 2010; Thierry et al. 2012). The gene is expressed in fetal and adult human brain, in particular in the adult cerebellum (Thierry et al. 2012), and is found as part of the spliceosome C (Chen et al. 2007). The phenotype of the patient is described in supplement S4.

## Discussion

In this study, we performed a follow‐up screen of candidate genes for EE in a new set of patients. After we had designed the project, EIEE26 and higher EEIE‐numbers were added to OMIM‐annotated genes for EIEE. Our experiment therefore lacks the EIEE‐genes *WWOX* (MIM 605131;EIEE28)*, AARS* (MIM 601065; EIEE29)*, SIK1* (MIM 605705; EIEE30) and *KCNA2* (MIM 176262; EIEE32). Among theEIEE genes in our design, likely pathogenic variants were found in recently discovered genes *GRIN2B* (MIM 138252;EIEE27), *EEF1A2* (MIM 602959; EIEE33) and *KCNB1* (MIM 600397; EIEE26). Our findings will help to expand on the phenotype descriptions associated with these genes, in separate publications.

In three unrelated patients, different novel variants in the gene *GPHN* were found, including one as a homozygote. This gene is described as showing recessive inheritance for various disorders, among which encephalopathy due to sulphite oxidase deficiency and Molybdenum Cofactor Deficiency (Reiss et al. 2001; Lionel et al. 2013). When comparing this number of novels in our data to the number of rare variants in ExAc as described, there was a relatively large excess in our data. Also, the gene is thought to be intolerant to LoF and missense variation (Samocha et al. 2014; Ware et al. 2015). Despite these observations, it seems unlikely that, these unique variants can by themselves explaining the disorder of the patient. Despite searching our data, we found no second possibly pathogenic variant in the heterozygote patients. Furthermore, the patient with a homozygote genotype had, according to the treating clinician, a phenotype that was not compatible with *GPHN* mutations.

Also surprising were a homozygote nonsense variant in *GRIN1* (Lemke et al. 2016) and a homozygote very rare missense variant in *GNAO1* (MIM 139311) rs758424351, both of which are described as genes with a dominant effect on EE. The phenotype of the patient with the *GNAO1* variant was not similar to phenotypes earlier described for dominant *GNAO1* mutations (Supp. S4) (Nakamura et al. 2013).

Seven patients in whom we found the variants that could probably explain their condition had related parents. Four of those had homozygous variants that we considered pathogenic, and one was compound heterozygous for a recessive gene. Of nineteen patients without related parents for whom we found the variants probably explaining their condition, none of the explaining variants was homozygous, but three patients were compound heterozygous for recessive genes. Though these observations are sparse for statistics, they are consistent with the idea that patients with related parents are more likely than patients with unrelated parents to suffer from a recessive form of EE (one‐sided *P *=* *0.01).

In support of other literature, our findings in X‐linked genes *IQSEC2* and *WDR45* question whether their description in OMIM as X‐linked dominant for EE‐like phenotypes is the only possible mode of action. In *IQSEC2,* variants were shown before to have an effect on intelligence in males and females (Gandomi et al. 2014; Tran Mau‐Them et al. 2014; Kalscheuer et al. 2015). In *WDR45,* loss‐of‐function variants are pathogenic in females, but in males are assumed to be incompatible with life (Haack et al. 2012). However, milder hemizygous variants may turn out to be variants of large effect on an ID or EE‐like phenotype in males.

We prioritized a number of presumed dominant‐acting candidate genes for follow‐up, and we Sanger‐sequenced patients' relatives for those genes if DNA was available (Table S1). Only an LoF variant in the candidate gene *HNRNPU* was *de novo,* so only for this gene we found supportive evidence for involvement in EE. Published information about this gene, combined with our own clinical data suggests a syndrome starting with early developmental delay, and followed by fever‐sensitive epileptic seizures within the first year of life. Later, afebrile seizures may develop. Developmental delay in described cases was severe, and various additional anomalies were mentioned (Need et al. 2012; Carvill et al. 2013; Epi4K‐Consortium et al. 2013; Hamdan et al. 2014) (https://decipher.sanger.ac.uk/ddd#research-variants/snvs, http://www.ncbi.nlm.nih.gov/clinvar/?term=hnrnpu[gene]). Phenotypic overlap may exist with patients with a 1q44 microdeletion encompassing this gene (Caliebe et al. 2010).

For all results, there is the caveat that we did not test whether the parental samples were from the true parents. For the patients with presumably recessive‐acting variants, all variants were shown to be inherited, but for *de novo* variants we have no guarantees.

### Prioritization

Our first tool to prioritize genes was calculating excess frequency of unique variants in our patients versus the population in the ExAC database. Because of differences in technology, huge differences in sample size, and imprecise estimate of coverage in both datasets, the size of the excess or *P*‐value may not be accurate. Yet, the fact that the ranking favored many known EE genes, even though most samples had been through more or less extensive diagnostic screening, shows that ranking based on excess of unique variants is a valid tactic.

Known EE genes not only showed on average an excess of unique variants in our patients relatively to the ExAC population, but also, on average, the unique variants in these EE‐genes in our patients were more deleterious, as assessed by the CADD‐scores, than those in the same genes in the ExAC population. Higher CADD‐scores were also found in genes annotated as ID‐genes or other related phenotypes (in particular *CDKL5* (MIM300203)*, IQSEC2, FARS2* (MIM 611592)*, CASK* (MIM 300172)*, POLG* (MIM174763)). Though some candidate genes also showed an excess of unique variants relative to the ExAC populations, these genes did not seem to have excessively high CADD‐scores on average in our patients for unique variants. So if high average CADD‐scores are an indication of gene pathogenicity, the high‐ranking candidate genes probably contained few true EE genes. It should be noted that CADD‐scores were only available for missense variants, while for example in *GLB1L3* two of the three unique variants were indels. Therefore, the contribution of loss‐of‐function variants due to frameshifts may be underestimated.

In a recent paper (Grozeva et al. 2015), a large cohort of patients with intellectual disability were sequenced without the parents for a set of candidate genes, as was done in this study. The authors also used enrichment of rare variants versus a control group as a criterion for pathogenicity of genes, though they had a stronger focus on LoF variants. They estimated that ~8% of their cohort had a pathogenic loss‐of‐function variant. While our dataset seems enriched for possibly pathogenic LoF‐variants (Table I, Supporting Information), the estimate for our EE patients will probably be lower.

Our cohort contained patients whose inclusion criteria contained seizures. In less selected cohorts of patients with developmental delay (e.g., Decipher Developmental Delay cohort (McRae et al. 2016)), it was shown that a considerable proportion of patients who have a presumed pathogenic variant in an epilepsy‐associated gene are described as having no seizures. Similarly, presumed pathogenic variants in intellectual disability or developmental delay associated genes are found in patients with seizures. The nonepilepsy genes in our design had been selected because they were reported with suspect variants in epilepsy patients before. Our analysis confirms that patients with presumed pathogenic variants in these genes (in Table S1 as “related” or “ID”) have been clinically interpreted as patients with epileptic encephalopathy, showing that there is considerable overlap. We found 13 presumed pathogenic DNMs in 29 genes with a dominant inheritance mode for EE, and 5 presumed pathogenic DNMs in 22 genes with a dominant inheritance mode for related phenotypes. This is in addition to recessively acting genotypes.

In the DDD paper, 23% of patients had a DNM in a known gene, which could explain the presence of the disorder. In our cohort, only ~6% of patients had an explaining DNM in a known gene. However, our sample was enriched for patients with presumably inherited pathogenic variants. If only considering the sporadic patients whose parents were not known to be related (*N* = 251), the proportion that could be explained by a DNM in a known gene was still only ~7%. Around 40 additional patients had variants in known EE or ID genes, but could not be followed up within the current project. Among those variants, a proportion will likely be explanatory for the patient's disorder. For some of the patients, the variants (dominant or recessive) that we identified in candidate genes, but could not follow‐up, will likely be the main cause of their disorder. Following up on all other identified variants, and screening the remaining patients for a wider set of epilepsy or developmental delay associated genes will likely increase the proportion of patients with a genetic explanation.

The limited number of variants that could be followed up, due to money constraints, unavailability of parental DNA and consent issues, is a limitation of this study. We selected the most promising candidate genes for follow‐up, and only found a single likely pathogenic variant. Yet, some other candidate genes may still turn out to harbour pathogenic mutations.

In conclusion, we found pathogenic variants in known EE genes, including recently identified epilepsy genes, but also in genes annotated for ID or other related phenotypes. We could not convincing show that any of the other candidate genes was a true EE gene. For *HNRNPU* we provided supportive evidence. Excess of loss of function variants suggests that a few more candidate genes may be real EE genes, but relatively low CADD‐scores for unique variants suggests that many of our candidate genes are not relevant for EE.

## Conflict of Interest

The authors declare to have no conflict of interest. Since mid‐October 2015, KH is under employment of UCB Pharma (Braine‐l'Alleud, Belgium). The company had no part in this study.

## Supporting information


**Table S1** Overview of all genes, the number of possibly pathogenic variants found and the number of variants tested in patient's relatives.

**Table S2** overview of all possibly pathogenic variants.

**Tables S3** Statistical tables for comparisons of CADD‐scores in various categories.

**Appendix S1** Phenotypic description patient with homozygous missense.

## Appendix 1 EuroEPINOMICS RES – working group collaborators

Balling R, Barisic N, Baulac S, Caglayan HS, Craiu DC, Depienne C, Gormley P, Hjalgrim H, Hoffman‐Zacharska D, Jähn J, Klein KM, Komarek V, LeGuern E, Lerche H, May P, Muhle H, Pal D, Palotie A, Rosenow F, Selmer K, Serratosa JM, Sisodiya SM, Stephani U, Sterbova K, Striano P, Talvik T, van Haelst M, Verbeek N, von Spiczak S, Weber YG.
