# Supplementary material for: Targeted sequencing of 351 candidate genes for epileptic encephalopathy in a large cohort of patients
Source: Mol Genet Genomic Med. 2016 Jul 30;4(5):568–80. doi: 10.1002/mgg3.235 (PMC5023942; doi:10.1002/mgg3.235)
Supplement: Supplementary file 3 — Tables S3 Statistical tables for comparisons of CADD‐scores in various categories. [file MGG3-4-568-s003.docx]

# Supplemental Tables S3.

Table CADD-scores for unique variants in our patients (TEGA) and the ExAC database for three categories of genes (1) EE genes, (2) genes involved in related phenotypes and (3) candidate genes.

| **Report** | | | | |
| --- | --- | --- | --- | --- |
| CADD_raw | | | | |
| phenotype_OMIM | set | Mean | N | Std. Deviation |
| candidate | exac | 3,34 | 58.746 | 2,52 |
|  | TEGA | 3,51 | 275 | 2,52 |
|  | Total | 3,34 | 59.021 | 2,52 |
| EE | exac | 3,50 | 3.817 | 2,41 |
|  | TEGA | 5,32 | 48 | 2,61 |
|  | Total | 3,52 | 3.865 | 2,42 |
| related | exac | 3,26 | 5.007 | 2,35 |
|  | TEGA | 4,76 | 35 | 3,29 |
|  | Total | 3,27 | 5.042 | 2,36 |
| Total | exac | 3,34 | 67.570 | 2,51 |
|  | TEGA | 3,88 | 358 | 2,69 |
|  | Total | 3,34 | 67.928 | 2,51 |

| **Tests of Between-Subjects Effects** | | | | | |
| --- | --- | --- | --- | --- | --- |
| Dependent Variable: CADD_raw | | | | | |
| Source | Type III Sum of Squares | df | Mean Square | F | Sig. |
| Corrected Model | 426,274^a^ | 5 | 85,255 | 13,753 | 1,88E-013 |
| Intercept | 10453,163 | 1 | 10453,163 | 1.686,294 | 0,00E+000 |
| set | 233,381 | 1 | 233,381 | 37,649 | 8,52E-010 |
| phenotype_OMIM | 187,539 | 2 | 93,770 | 15,127 | 2,70E-007 |
| set * phenotype_OMIM | 139,428 | 2 | 69,714 | 11,246 | 1,31E-005 |
| Error | 411941,530 | 66.454 | 6,199 |  |  |
| Total | 1141966,611 | 66.460 |  |  |  |
| Corrected Total | 412367,804 | 66.459 |  |  |  |
| a. R Squared = ,001 (Adjusted R Squared = ,001) | | | | | |

CADD-scores for unique variants in known EE genes, which were classified as either pathogenic or benign after investigating relatives and patient records.

Ben_dom: benign in dominant gene, ben_rec: benign in recessive gene, path_dom: pathogenic in dominant gene, path_rec: pathogenic in recessive gene.

| **Report** | | | |
| --- | --- | --- | --- |
| CADD | | | |
| bin_result | Mean | N | Std. Deviation |
| ben_dom | 3,726 | 8 | 2,021002992 |
| ben_rec | 1,265 | 1 | . |
| path_dom | 7,069 | 18 | 2,395142071 |
| path_rec | 5,405 | 5 | ,508449945 |
| Total | 5,792 | 32 | 2,614559955 |

| **Tests of Between-Subjects Effects** | | | | | |
| --- | --- | --- | --- | --- | --- |
| Dependent Variable: CADD | | | | | |
| Source | Type III Sum of Squares | df | Mean Square | F | Sig. |
| Corrected Model | 148,682^a^ | 5 | 29,736 | 12,227 | 3,696E-006 |
| Intercept | 359,374 | 1 | 359,374 | 147,770 | 3,146E-012 |
| bin_result | 96,503 | 2 | 48,252 | 19,840 | 5,861E-006 |
| inheritancegene | 34,779 | 1 | 34,779 | 14,301 | 8,241E-004 |
| bin_result * inheritancegene | 1,347 | 1 | 1,347 | ,554 | ,463 |
| Error | 63,231 | 26 | 2,432 |  |  |
| Total | 1285,384 | 32 |  |  |  |
| Corrected Total | 211,914 | 31 |  |  |  |
| a. R Squared = ,702 (Adjusted R Squared = ,644) | | | | | |
