# Supplementary material for: Targeted sequencing of 351 candidate genes for epileptic encephalopathy in a large cohort of patients
Source: Mol Genet Genomic Med. 2016 Jul 30;4(5):568–80. doi: 10.1002/mgg3.235 (PMC5023942; doi:10.1002/mgg3.235)
Supplement: Supplementary file 4 — Appendix S1 Phenotypic description patient with homozygous missense. [file MGG3-4-568-s004.docx]

# Patient with *HNRNPU* *de novo* p.Val604Val-fs24* mutation

Female, presenting with febrile seizures at 8 months. Developmental delay was noted before the first seizures, and at age two, she did not speak. There were deep central matter deviations on MRI, possible indicative of delayed myelination. EEG was epileptiform. Other characteristics are hyperlaxity and hypotonia. She has deep-set eyes with epicanthal folds and bags below the eyes, a narrow palate, grey sclerae, short 2nd digit on both hands, palmar crease on the left hand. Normal stature. She has small ears, like her mother.

# Patient with rs758424351 (*GNAO1)* homozygote missense mutation

Female, born in 1999, normal pregnancy and delivery, development was normal too. At age 9 years she had a high fever 40 c without seizure and she was discharge from the hospital without treatment, but she was weak. Four days later during the day she showed loss of consciousness for one minute without any seizures. The parents brought her to the hospital. In the hospital she started to convulse, one convulsion after the other (status) without stop for 3 days. She was moved to another hospital where she continued to convulse without stop for 5 weeks. They tried to anesthetize her twice. She did not respond to all the medication. Then she went to rehabilitation for 8 months. During this time, she had some seizures, but not many. Type of the seizure: GTCS, salivation, rotation of the eyes in back, loss of consciousness for one minute, loss of urination. Post-ictal EEG – abnormal. At age of 10 years she had the last seizure. Now she is studying in a normal school. There is a mild cognitive and motoric delay. She stopped taking the medication (Tegretol and Frisium) the last year.

*This patient does in retrospect not meet our inclusion criteria.*
